# Human-Terrestrial Wildlife Conflict in Ethiopia: A Systematic Review

**DOI:** 10.1155/2022/2612716

**Published:** 2022-08-29

**Authors:** Getahun Shanko, Bekele Tona

**Affiliations:** Department of Natural Resource Management, Wolaita Sodo University Dawro Tarcha Campus, P. O. Box 01, Tarcha, Ethiopia

## Abstract

We conducted a review of 26 articles published between 2009 and 2021 to determine human-wildlife conflict based on spatial and temporal patterns, biological components, drivers of conflict, and mitigation methods used. We employed search, synthesis, appraisal, and analysis framework for review and VOSviewer software for network analysis. We included articles that only focused on relations between terrestrial wildlife and humans, while others deal with ecology, distribution, and biology of wildlife because it does not go with HWC. Forty-seven species of terrestrial vertebrates were reported in conflict-related studies, being Bovidae and Cercopithecidae the most frequently studied groups, of which eleven are found in threatened list species. The main drivers reported were land use change, proximity to protected areas, and illegal resource exploitation. In the management case, the use of traditional protection techniques such as fencing, guarding, and physical barriers was reported. About 178 keywords' analysis revealed a focus on “coexistence,” “mitigation,” and “food security.” The literature focused mainly on larger mammals, led by Ethiopian authors, and excluded the social dimensions of HWC. Therefore, identifying conflict-prone species focuses on the social dimensions of coexistence, such as human attitudes towards terrestrial wildlife, and broadening the taxonomic and cultural breadth of HWC is required.

## 1. Introduction

The conflict between humans and wildlife is a global issue, happening both in developing and developed countries [1]. Human-wildlife conflict (HWC) is a serious global issue in the developing world where expansion of settlements and human population growth are reducing wildlife habitats and increasing HWC [2]. Crop cultivation and livestock raring in developing countries are the main sources of rural income and livelihood [3, 4] which shirk the habitat of wildlife and lead finally to HWC.

In addition, HWC arises mainly because of fragmentation, degradation, and loss of habitats through human activities such as agricultural expansion, settlement, and animal husbandry [5]. A failure of conservation practices of wildlife is also an important factor for HWCs in this world [6]. HWC is also regarded as a serious challenge for wildlife management [7]. It led to disruption of psychology and economic development, the spread of diseases, and raised the extinction spectrum [8, 9].

Currently, effective management of wildlife should have an objective of managing the relationship between people and wildlife to achieve the desired goals stated by different stakeholders [10]. It also requires policymakers and conservation managers to take into consideration that the support and cooperation of stakeholders are required to meet the conservation goals [11]. Mitigation of HWC is central to ecosystem health and human safety; however, this requires an insightful consideration of interrelated ecological and social relations [12]. Therefore, understanding stakeholder's attitude living near to wildlife is recognized as an essential to design and implement a successful HWC mitigation measure [11].

Drivers of environmental change such as a change in land use, poaching, species introductions, climate change [13], and indirect drivers like change in structures of governance, demography, economy, and culture play important roles in shaping human-wildlife relations. Competition between domestic animals and wild animals for food prey [14] is also another cause of HWC in natural environments. Therefore, HWCs are considered a major threat to world economic development and biodiversity conservation [15].

Over the last decade, the research on coexistence and conflicts between wildlife and humans has grown exponentially in different forms like reports and articles [7, 9]. According to the study in Hindu Kush Himalaya, there was a 57% increase in publications in the last decade, but with a disproportionate geographical focus [7]. Similarly, in the Asian countries of Indonesia, Nepal, and India, 87% of the publications concentrated on HWC [16] because the region accounts for high biological diversity, with high threat due to overexploitation and agricultural expansion [17]. Similarly, 87% of publications on HWC are from Africa and Asia and a 92% increase in South America from 2000 to 2015 [18]. However, some countries like Southeast Asia, the Middle East, Central America, Central Europe, and most African countries get less attention [19].

To mitigate HWC, local people use chemical repellents, lethal control of problematic individuals, fertility control, and change animal behavior using provocative stimuli [20], understanding the ecological and spatial dynamics of human-wildlife [16] among others. If its effectiveness is proved, it is important to use nonlethal methods for mitigating HWC since it has both ecological and economic values for maintaining and conserving the species.

In countries like Ethiopia, HWCs are more vulnerable because livestock production and agricultural practices are an important part of human livelihoods [21, 22]. Thus, investigating the conflicts between wildlife and human populations has become a relevant scientific work since it allows us to see the pattern in the conflict occurrence and identify different aspects that can increase human's tolerance during such conflicts.

Therefore, we conducted a literature review on HWC in Ethiopia based on published articles between 2009 and 2021. The study aimed to identify terrestrial wild animal species involved in conflicts with humans on a country-level and to investigate their occurrence. Thus, the present study aims mainly to evaluate the conflict in taxonomic terms, the types of HWC incidences experienced by people, methods used to mitigate HWC, and analyze the joint network of research by studying their keywords for a better understanding of the knowledge gaps, priorities, and trends in the research.

## 2. Methods

**Figure 1:**
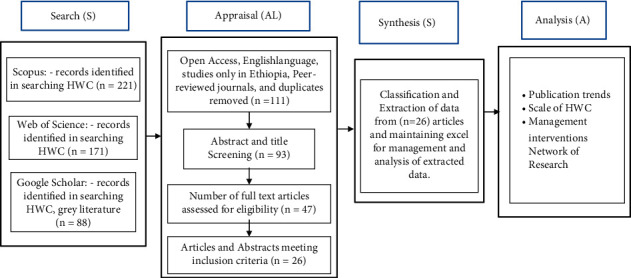
Flow diagram using SALSA framework.

The aim of a systematic literature review (SLR) is to summarize the results from large research outputs [23]. We employed the SLR approach of qualitative content analysis. We used the Grant and Booth [24] framework that involves four major steps, as shown in Figure 1: search (database type with defined searching string), appraisal (literature exclusion and inclusion, and criteria for quality assessment), synthesis (extraction and data categorization), and analysis (narrative and conclusion) (SALSA). The method is systematic, accurate, reproducible, and exhaustive [25, 26].

### 2.1. Search

This phase is crucial to determine the important databases to gather relevant documentation using an appropriate search string [27]. We used Scopus, Google Scholar, and Web of Science as search databases. Both Scopus and Web of Science have more indexed journals and are the largest databases [28]. Google Scholar was used to including citations that are not incorporated into other databases [29]. We searched the Web of Science and Scopus databases by using a search string that included two basic elements, human-wildlife relations and conflicts.

We used “conflict” search terms (wildlife ^*∗*^ OR vertebrate OR herbivore OR carnivore ^*∗*^ OR felid ^*∗*^ OR canid ^*∗*^ OR fauna OR primate OR monkey) AND (attack ^*∗*^ OR conflict ^*∗*^ OR harm ^*∗*^ OR impair ^*∗*^ OR damage ^*∗*^ OR raid ^*∗*^ OR crop ^*∗*^ OR livestock ^*∗*^ OR cattle OR predation ^*∗*^ OR depredation ^*∗*^ OR retaliation OR dead OR death OR mortality OR lethal) AND (Ethiopia OR Abbay OR Blue Nile OR Baro-Akobo OR Setit-Tekeze OR Atbara OR Mereb OR Awash, Denakil OR Omo-Gibe OR Central Lakes OR Wabi-Shebelle OR Genale-Dawa OR Ogaden OR Gulf of Aden OR Afar OR Amhara OR Benishangul-Gumuz OR Gambela OR Harari OR Oromia OR Sidama OR Somali OR South West OR Southern Tigray OR Addis Ababa OR Dire Dawa and Coexistence” search terms (wildlife ^*∗*^ OR vertebrate OR herbivore OR carnivore ^*∗*^ OR felid ^*∗*^' OR canid ^*∗*^ OR fauna OR primate OR monkey) AND (avoid ^*∗*^ OR solution ^*∗*^ OR coexistence ^*∗*^ OR manage ^*∗*^ OR prevent ^*∗*^ OR nonlethal OR non-lethal) AND (Ethiopia OR Abbay OR Blue Nile OR Baro-Akobo OR Setit-Tekeze OR Atbara OR Mereb OR Awash, Denakil OR Omo-Gibe OR Central Lakes OR Wabi-Shebelle OR Genale-Dawa OR Ogaden OR Gulf of Aden OR Afar OR Amhara OR Benishangul-Gumuz OR Gambela OR Harari OR Oromia OR Sidama OR Somali OR South West OR Southern Tigray OR Addis Ababa OR Dire Dawa). The search query was used to include all published articles for the past 13 years (2009–2021). English language articles and studies carried out only in Ethiopia were used for this study. Title, abstract, and keywords fields were applied to the search engine [19].

### 2.2. Appraisal

In this phase, the selected articles were evaluated based on their objective. We limited the search to peer-reviewed articles that were published in English, studied only in Ethiopia, and removed the duplicates and this yielded 111 articles. We then screened abstracts and titles to confirm that we only incorporated empirical studies (i.e., conceptual papers and reviews were excluded). In addition, we only included articles that focus on relations between terrestrial wildlife and humans and articles that allowed the identification of the type of conflict, i.e., crop damage, predation of domestic animals, and attack, injury, or death of humans. We excluded articles that focus on ecology, distribution, and biology of wildlife because it does not go with HWC, and articles that focused on the aquatic ecosystem were also excluded since our study deals with a terrestrial ecosystem which yielded 93. A total of 26 articles fulfilled the eligibility criteria (only articles that show the types of conflicts considered as domestic animal predations, crop damage, and attack, death, or injury of human beings).

### 2.3. Synthesis

Information extraction and classification from selected articles to derive conclusions and knowledge were consisted in this step. From the selected articles, information is extracted by the authors using the prepared criteria [30]. For processing, the data were managed and maintained in Microsoft Excel.

### 2.4. Analysis

Under this phase, evaluate synthesized data to get meaningful information based on the selected papers. The study used VOSviewer (https://www.vosviewer.com) software for visualizing and constructing bibliometric networks [31]. We visualized keyword frequency to analyze areas where the most researched areas related to our title. The map formed in the VOSviewer incorporated keywords connected by lines. The strength of a link indicates the number of publications where similar keywords occurred.

## 3. Results

### 3.1. Spatial and Temporal Pattern

**Figure 2:**
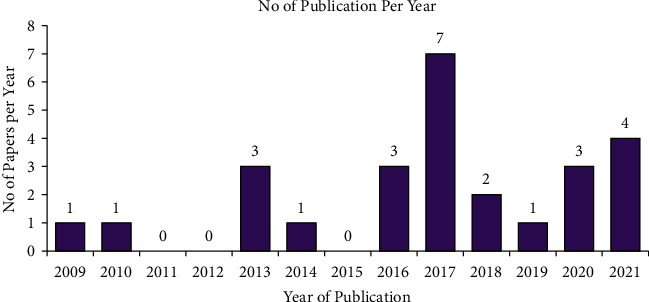
Temporal distributions of studies on HWC in Ethiopia.

Since 2009 (one article), the number of studies published in international journals increased, as in 2017, seven articles (figure 2). Most studies (80.8%, *n* = 21 articles) involved more than one species and were involved in HWC. Publications involving a single animal [32, 33] were less frequent than those containing multiple wild animals, about more than ten [34]. Geographically, out of eleven regions and two city administrations in Ethiopia, the research was conducted in Oromia (46.2%, *n* = 12), Southwest Ethiopia (23.1%, *n* = 6), Amhara (11.5%, *n* = 3), SNNPR (7.7%, *n* = 2), and Gambela, Tigray, and Afar which share 3.85% (*n* = 1) each.

### 3.2. Biological Components

**Figure 3:**
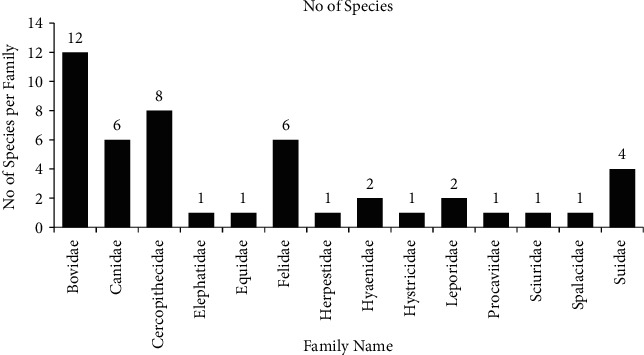
Distribution of taxonomy in reviewed studies.

A total of 47 wild terrestrial species of mammals distributed among eight orders and fourteen families were recorded as target species of conflicts in Ethiopia. Order Artiodactyla (represented by 16 species) is followed by order Carnivora (includes 15 species). Order porboscidea, perissodactyla, and andhyracoidea are represented by single species. The five families with the highest number of conflictual species were Bovidae (*n* = 12), Cercopithecidae (*n* = 8), Canidae (*n* = 6), Felidae (*n* = 6), and Suidae (*n* = 4) (Figure 3).

**Figure 4:**
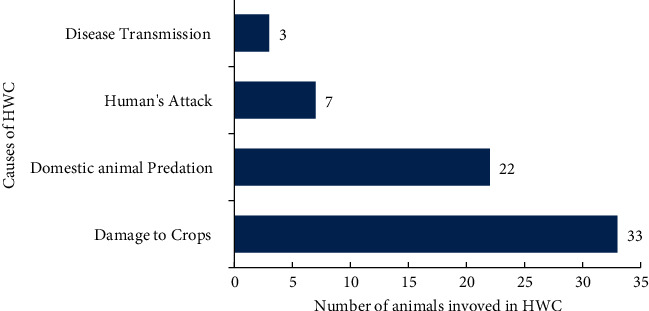
Terrestrial wildlife species involved in HWC.

Of the wildlife's, 33 species were cited for crop damage and 21 for domestic animal predation, 7 for human's attack, and 3 for disease transmission (Figure 4). The five species frequently reported as problematic in the articles were olive baboon (*Papio Anubis*, 21 studies), porcupine (*Hystrix cristata*, 17 studies), spotted hyena (*Crocuta crocuta*, 15 studies), leopard (*Panthera pardus*, 12 studies), and common warthog (*Phacochoerus africanus*, 10 studies).

Different wild animals were recorded in at least two types of conflict. Two species, namely, mongoose (*Helogale hirtula*) and gelada baboons (*Theropithecus gelada*) were recorded for both crop damage and domestic animals predation conflicts; lions (*Panthera leo*) were recorded for both human attacks and domestic animals predations conflicts [35]; African Savanna elephant (*Loxodonta africana*) for livestock predation, crop damage, and human attacks [36]; African buffalo and Olive baboon recorded all four types of conflicts. Out of the total 47 species recorded, eleven were found in the IUCN Red List [37] of Threatened Species (Table 1). Three species are listed as Vulnerable, three as Near Threatened, and five as Endangered.

### 3.3. Drivers for the Conflict

**Figure 5:**
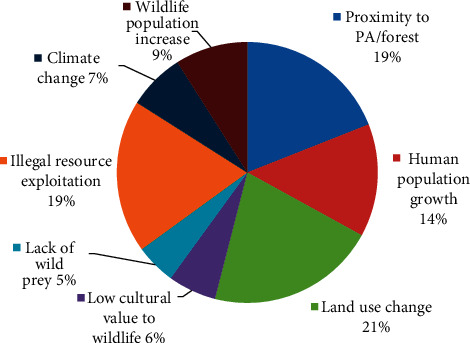
Drivers of HWC-related change considered by articles expressed in percentages.

More than 75% (*n* = 20) of the reviewed articles considered more than one driver that triggers HWC in Ethiopia (Figure 5). The most common drivers mentioned in the articles were land use change (23%, *n* = 6), particularly agriculture; the proximity of community settlements to protected areas (PA) or forest (19%, *n* = 5); illegal resource exploitation (19%, *n* = 5) such as illegal poaching and illegal grass collection; and human population growth (15%, *n* = 4), mainly encroachment. The others included were wildlife population increase (11%, *n* = 5), climate change (8%, *n* = 2) due to natural and anthropogenic factors, and low cultural value (8%, *n* = 2) for wildlife such as lions and lack of wild prey (8%, *n* = 2) as a result of the destruction of forests, which were a significant driver of HWC in Ethiopia.

### 3.4. Management Intervention

**Figure 6:**
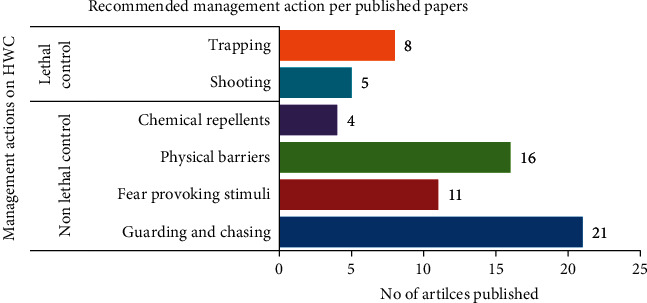
Management actions on HWC recommended by research articles.

As management strategies, about 88.5% (*n* = 23) of the reviewed literature mentioned and recommended nonlethal and lethal control (Figure 6); and most of these studies recommended more than two management actions simultaneously. Nonlethal measures were the most commonly considered in alleviating conflicts between humans and wildlife. Thus, 80.7% (*n* = 21) of articles considered guarding and chasing their crops and livestock by deploying watchdogs and aided by smoke fire; 65.4% (*n* = 17) of articles considered physical barriers such as building wooden bomas, fencing, and water towers; 42% (*n* = 11) of articles mentioned fear-provoking stimuli like scarecrows and beating drums; and chemical repellents such as strange scents were used as nonlethal methods to end the conflict. Lethal control methods like shooting and trapping were also considered to mitigate conflict.

### 3.5. Co-Occurrence of Keywords in HWC in Ethiopia

**Figure 7:**
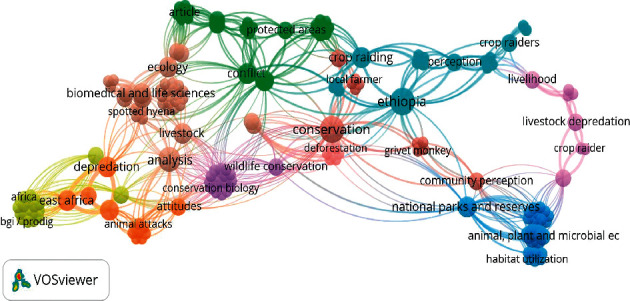
Network of keywords co-occurrence for HWC research in Ethiopia.

A total of 178 keywords were found in the reviewed literature, of which 136 appeared only once. Some of them include “coexistence,” “mitigation” “food security,” and “livestock husbandry” (Figure 7). The most frequently (5 times) occurred keywords were “Ethiopia,” and “conservation,” followed by “analysis” (4 times). As compared to keywords with low occurrence, there is high link strength for those frequently occurred keywords. “Livestock,” “ecology,” “agriculture” and “national parks” were among the top keywords with the highest link strength.

## 4. Discussion

Since 2009, the number of published papers dealing with HWC has increased. This is mainly related with an increased number of wildlife populations [38, 39] and the proximity of settlements and farmland to wildlife habitats [32, 39–41]. Meanwhile, there was an overstocking rate of livestock, which made them vulnerable to attacks by wildlife [42]. In addition, the relations between small or middle-sized animals and humans, like the Hystricidae, Herpestidae, and Procaviidae families, have received less attention.

The impact of HWC may vary according to conflict types and the degree of tolerance by humans. People do not want to tolerate animals that kill their livestock and humans. Various human-wildlife relations occur that are highly tied to local communities and indigenous lifestyles, in various ways. For example, in Kaffa highland between 2009 and 2013, about 350 livestocks were attacked by lions, 62 were attacked by leopards, and about 12 attacks on people, but they tolerate it, and also, there is deep-rooted respect and honour for lions maintained even at the time of attacks [43]. Others like [34], wildlife was conserved due to ethical values, maintenance of ecosystem balance, enjoyment from viewing wildlife, and source of income to the local people. By contrast, in Gambella, the attack of wildlife on humans and livestock was much less than in Kaffa highland, but they do not want to increase the population of lions even in national parks, and if it is allowed by the law, they want to kill because of retaliation [43].

The review also establishes that most of the HWC studies done in Ethiopia have focused on the damage caused to both livestock and crops by wild animals. In this way, it was found that in Meskan district of the Gurage zone, there incurred an annual loss of maize of around 42% due to crop raids by foraging animals [44] and about 27.96% of livestock due to depredation [34]. Such losses pose a challenge to the country's local economy and food security.

The review also described that most livestock attacks occurred at night, with the reason of lack of efficiency in prevention; and during the wet season, with the reason of flooding of plains and migration of the prey to neighboring countries [43]. About 23.4% of the species compiled in this review are found on the IUCN's list of threatened species. When we examined keywords, the conflict featured with associated species and type of damage including protected areas.

The results of this review also revealed that illegal poisoning affected *Canis simensis* [45], which was an endangered species according to IUCN. Different nonlethal methods that are used to reduce HWC are guarding [33, 40, 41] with the help of dogs through chasing livestock predators [38], physical barriers like fencing [39, 46], loud noises [47], seasonal displacement [33], fear-provoking stimuli such as scarecrows and beating drums [48, 49], sound mechanisms for scaring [44], and repellent chemicals like scents [34]. Lethal methods used are shooting and trapping [34, 50].

It also pointed out that poultry are rarely attacked by wild animals, mainly white-tailed mongoose (*Ichneumia albicauda*), and due to this, they are used for chasing and guarding [38]. Another important way of minimizing the impact of HWC is through the provision of compensation for the losses arising from the conflicts to improve attitudes toward wildlife and enhance their survival [33]. However, compensation is not considered the best way to reduce HWCs.

### 4.1. Implication for Conservation

Conflicts between humans and wild animals are recognized as major issues in conservation of wildlife species. Different types of encroachment in areas adjacent to protected areas in Ethiopia have been widely documented. Our review evaluated the trend, status, biological components, drivers, and management intervention of HWC findings in Ethiopia. In order to foster human-wildlife coexistence, different individuals must understand the benefits and costs of wildlife. The result showed an increase in interest in studying HWC; however, the research is biased geographically. Most of the studies focused on nationally significant PAs and surrounding. But conflict management demands greater emphasis at a large scale level since the habitat of wildlife goes beyond it. Studies on each species that causes HWC and the impact it causes should be quantified to measure the degree of conflict. The nonlethal method to manage HWC should be widened since it allows biodiversity conservation.

## Figures and Tables

**Table 1:** Wild species listed in IUCN.

Species		Types of conflicts				Status IUCN	Number of articles that recorded the species
Common name	Scientific name	Crop damage	Livestock predation	Human attack	Disease transmission		
Leopard	*Panthera pardus*		x			Vulnerable	12
Lion	*Panthera leo*		x	x		Vulnerable	6
Cheetah	*Acinonyx jubatus*		x			Vulnerable	1
Gerenuk	*Litocranius walleri*	x				Near threatened	1
Striped hyaena	*Hyaena hyaena*		x			Near threatened	1
Swayne's hartebeest	*Alcelaphus buselaphus* ssp. *swaynei*	x				Endangered	2
African wild dog	*Lycaon pictus*		x			Endangered	2
Grevy's zebra	*Equus grevyi*	x				Endangered	1
Ethiopian wolf	*Canis simensis*		x			Endangered	1
African buffalo	*Syncerus caffer*	x	x	x	x	Near threatened	5
African Savanna elephant	*Loxodonta africana*	x	x	x		Endangered	6

## Data Availability

The data used to support this study are available from the corresponding author upon request.
